# miRNA Genes of an Invasive Vector Mosquito, *Aedes albopictus*

**DOI:** 10.1371/journal.pone.0067638

**Published:** 2013-07-01

**Authors:** Jinbao Gu, Wanqi Hu, Jinya Wu, Peiming Zheng, Maoshan Chen, Anthony A. James, Xiaoguang Chen, Zhijian Tu

**Affiliations:** 1 Key Laboratory of Prevention and Control of Emerging Infectious Diseases of Guangdong Higher Education Institutes, Department of Pathogen Biology, School of Public Health and Tropical Medicine, Southern Medical University, Guangzhou, Guangdong, P.R. China; 2 Department of Biochemistry, Virginia Tech, Blacksburg, Virginia, United States of America; 3 Beijing Genomics Institute, Beishan Road, Shenzhen, Guangdong, P.R. China; 4 Departments of Microbiology & Molecular Genetics and Molecular Biology & Biochemistry, University of California Irvine, Irvine, California, United States of America; Kansas State University, United States of America

## Abstract

*Aedes albopictus*, a vector of Dengue and Chikungunya viruses, is a robust invasive species in both tropical and temperate environments. MicroRNAs (miRNAs) regulate gene expression and biological processes including embryonic development, innate immunity and infection. While a number of miRNAs have been discovered in some mosquitoes, no comprehensive effort has been made to characterize them from different developmental stages from a single species. Systematic analysis of miRNAs in *Ae. albopictus* will improve our understanding of its basic biology and inform novel strategies to prevent virus transmission. Between 10–14 million Illumina sequencing reads per sample were obtained from embryos, larvae, pupae, adult males, sugar-fed and blood-fed adult females. A total of 119 miRNA genes represented by 215 miRNA or miRNA star (miRNA*) sequences were identified, 15 of which are novel. Eleven, two, and two of the newly-discovered miRNA genes appear specific to *Aedes*, *Culicinae*, and *Culicidae*, respectively. A number of miRNAs accumulate predominantly in one or two developmental stages and the large number that showed differences in abundance following a blood meal likely are important in blood-induced mosquito biology. Gene Ontology (GO) analysis of the targets of all *Ae. albopictus* miRNAs provides a useful starting point for the study of their functions in mosquitoes. This study is the first systematic analysis of miRNAs based on deep-sequencing of small RNA samples of all developmental stages of a mosquito species. A number of miRNAs are related to specific physiological states, most notably, pre- and post-blood feeding. The distribution of lineage-specific miRNAs is consistent with mosquito phylogeny and the presence of a number of *Aedes*-specific miRNAs likely reflects the divergence between the *Aedes* and *Culex* genera.

## Introduction

MicroRNAs (miRNAs) are ∼22 nucleotides (nt) in length and modulate gene expression by targeting cognate mRNAs for cleavage or translational repression. miRNAs are distributed widely in metazoans and plants and are involved in the regulation of many biological processes including apoptosis, cancer, embryonic development, immunity and infection [1]–[3]. miRNAs also may have a role in mosquito responses to infection by malaria parasite and arboviruses [4], [5]. Many miRNAs are transcribed from independent promoters to generate the primary miRNAs (pri-miRNAs), which could contain one or more regions of complementary bases that form secondary structures (hairpins). These hairpins are recognized and liberated by the Microprocessor complex to make precursor miRNAs (pre-miRNAs). Pre-miRNAs are exported to the cytosol and processed further by Dicer to make the ∼22-nt miRNA:miRNA* duplex. The duplex is separated by a helicase and the single-stranded miRNA is loaded into the RISC complex., The miRNA* strand is normally rapidly degraded [6]. Other miRNAs reside in introns of genes and their biogenesis is independent of the Microprocessor complex [7], [8].

Deep-sequencing coupled with bioinformatic analyses has produced comprehensive catalogs of miRNAs in model organisms. For example, there are 171 miRNAs in *Drosophila melanogaster* discovered in a large number of samples derived from different developmental stages and tissues and from analysis of 12 *Drosophila* genomes [9], [10], [11]. Deep-sequencing efforts to uncover miRNAs in mosquitoes have been limited to specific developmental stages or cell lines and the number discovered is relatively low compared to *D. melanogaster*. A small number of miRNAs were verified experimentally in two *Anopheles* mosquito species [4], [12]. Ninety-eight miRNA genes that produce 86 distinct miRNAs were discovered in *Aedes aegypti*, mostly by small-scale 454 sequencing of embryo and midgut samples [13]. A total of 65 and 77 miRNAs were discovered following Illumina sequencing of small RNAs from an *Ae. albopictus* cultured cell line and blood-fed *Culex quinquefasciatus* females, respectively [5].While most of these miRNAs are conserved across divergent species, 11 distinct miRNA genes are found only in mosquitoes, some of which are restricted to certain taxonomic groups [5], [13].

Here we report discovery of 119 miRNA genes through deep-sequencing of small RNAs isolated from multiple developmental stages of *Ae. albopictus*, of which 15 are novel and appear to be only in mosquitoes. We chose *Ae. albopictus* for this study because of its rapid expansion in world-wide distribution and its emerging importance as a vector for Dengue and Chikungunya viruses [14]. Our analysis has doubled the number of known mosquito-specific miRNAs, uncovered miRNAs showing stage-specific and blood-meal-regulated expression profiles, and provided the basis for the investigations of the function and evolution of mosquito miRNAs.

## Materials and Methods

### Ethics Statement

All vertebrate animals were housed and handled in strict accordance with the guidelines of the institutional and national Committees of Animal Use and Protection. All experimental procedures on mice were approved by the Committee on the Ethics of Animal Experiments of Southern Medical University (Permit Number: SCXK 2006-0015).

### Mosquitoes

The CDC (Guangdong, China) strain of *Aedes albopictus* originated in Guangzhou, Guangdong province, PRC, and was established the laboratory in 1981. All mosquitoes were maintained in humidified incubators at 25±1°C on a 12 hour light:dark cycle.

### *Aedes albopictus* Sample Preparation for Illumina Sequencing

Embryos were collected 0–24 hours after egg deposition by placing a damp collection cup within a cage. Larval samples were collected at each instar and combined. Pupal samples were collected from a pool of varied ages. Male and female adults were collected five days post-emergence. Three- to five-day old adult females were fed on mouse blood and collected at 1, 3 and 5 days after feeding and pooled. Total RNA was isolated using Trizol (Invitrogen). Approximately 20 µg of the total RNAs were separated on a 15% denaturing polyacrylamide gel and small RNAs ranging up to 30 nt in length were excised and sent to the Beijing Genome Institute Inc. for sequencing and analysis. The small RNAs were ligated sequentially to 5′- and 3′-end RNA adapters. The small RNA molecules were amplified for 17 cycles using the adaptor primers and fragments ∼90 nt in length (small RNA+adaptors) were isolated from agarose gels. The purified DNA was used directly for cluster generation and sequencing analysis using the Illumina Genome Analyzer (Illumina, San Diego, CA, USA). Clean reads were processed for computational analysis after removing adaptor sequences and contaminated reads. ). All primary sequence read data have been submitted to the National Center for Biotechnology Information (NCBI) short-read archive (accession number SRA060684).

### Bioinformatics

After removing low-quality sequences determined by inspection of chromatographs, tags with lengths ranging from 18–30 nt were selected for further analysis. The subsequent procedures performed with Solexa were summarizing data production, evaluating sequencing quality, calculating length distribution of small RNA reads and filtering reads contaminated by rRNA, tRNA, snRNA, snoRNA, repeat, exon and intron sequences using the NCBI Genbank database (http:www.ncbi.nlm.nih.gov/). *Aedes aegypti*, a closely related mosquito in the same subgenus (Stegomyia) as *Ae. albopictus,* provided a valuable “reference genome” to which clean reads were aligned using SOAP [15]. Sequences with a perfect match or one mismatch were retained for further analyses. RNA secondary structures were analyzed using 100 nt of genomic DNA flanking each side of the sequence, and the secondary structures predicted using RNAfold (http://rna.tbi.univie.ac.at/cgi-bin/RNAfold.cgi) and analyzed by MIREAP (http://sourceforge.net/projicts/mireap) at default settings. MIREAP is designed specifically to identify genuine miRNAs from deeply-sequenced small RNA libraries. It considers miRNA biogenesis, sequencing depth and structural features to improve the sensitivity and specificity of miRNA identification. Stem-loop hairpins were considered typical only when they fulfilled three criteria: mature miRNAs are present in one arm of the hairpin precursors, which also lack large internal loops or mismatches; the secondary structures of the hairpins are stable with free energies of hybridization lower than -20 kcal/mol; and hairpins are located in intergenic regions or introns. Those genes whose sequences and structures satisfied all of these criteria were considered candidate miRNA genes. Subsequently, the computational approach, miRAlign, was adopted to predict new miRNA genes that are paralogues or orthologues to known miRNAs sequences from miRBase [16]. The final step of miRNA confirmation was performed by aligning Illumina small RNA reads to the predicted pre-miRNA secondary structures to rule out any potential false positives following the stringent criteria described in Berezikov *et al.* [17]. *Aedes albopictus* miRNAs were compared with the miRBase version 17 using Blast searches with a low stringent cutoff (e-value 10 and window size 7) to ensure that we do not miss any potential homologues in miRBase (v.17). BLAST hits then were subject to manual inspections. In addition, Mapmi pipeline [18] was used to find homologues in the available genome assemblies of other insect species including *Ae. aegypti*, *C. quinquefasciatus*, *An. gambiae*, and four *Drosophila* species, *D. melanogaster, D. ananassae, D. pseudoobscura*, and *D. grimshawi* [19]. Two settings were used, one allowing only one mismatch and the other allowing three mismatches, and the results compared. We use 35 as the Mapmi cutoff score for homologues [18]. Potential homologues with a score of less than 35 are subjected to further analysis by miRscan, which takes into account evolutionary conservation [20]. Read counts of each miRNA in the six samples were obtained as described previously [13]. Either the mature miRNA or miRNA* should have at least 15 reads in the six samples for a miRNA gene to be considered expressed. All expression data were normalized with the following formula: Normalized Expression of a miRNA =  (The count of the miRNA in a particular sample)/(Total miRNA counts from this sample)×1 million. A hierarchical clustering analysis with MeV 4.8 using Pearson correlation with average linkage was used to evaluate the expression pattern of individual miRNAs [21], [22]. Prediction of miRNA targets was performed using miRanda (version 3.3a) [23]. The 3′-end UTRs retrieved from Vectorbase (transcripts version L1.2) were used as an input and the miRanda cut-off score was set at 150. The GO terms of the predicted targets were retrieved from Vectorbase Biomart. The GO terms of 6741 out of 9726 transcripts that have annotated 3′-end UTR also were retrieved from Biomart and used as reference in GO enrichment test. The GO enrichments for targets of each miRNA cluster were performed by blast2go using Fisher’s exact test under a false discovery rate of 0.01.

### Northern Blots

Unless noted otherwise, sample collection conditions for northern blot analyses were identical to those described for preparing samples for Illumina sequencing. Larvae were collected at each instar and pooled to generate early (I and II instars) and late (III and IV instars) larval samples. Female adults were fed on mouse blood and sugar water, and blood-fed samples were collected at 24hrs post-blood-meal. Samples were either homogenized immediately for RNA isolation or flash frozen in liquid nitrogen immediately following collection, then stored at –80°C. Total RNA isolation was carried out using a mirVana miRNA isolation kit (Ambion, Austin, TX). Northern blots were carried out based on Mead *et al.* [12]. Briefly, total RNA was loaded onto 15% denaturing polyacrylamide gels, and run with ssDNA markers 19 and 23 nt in length. The RNA gels were transferred to BrightStar-Plus nylon membranes (Ambion), cross linked with UV, prehybridized, and then hybridized overnight in the ULTRAhyb-Oligo Hybridization Buffer (Ambion) with the appropriate DIG-labeled probe at 42°C. Wash conditions were the same as described in Mead *et al.* [12]. Antisense 5′ digoxigenin-labeled miRCURY LNA probes were purchased from Exiqon (Vedbaek, Denmark). Probe sequences were complementary to those shown in Li *et al*. [13].

## Results and Discussion

### Small RNA Sequencing and Evidence for Transcription of Known miRNAs

**Figure 1:**
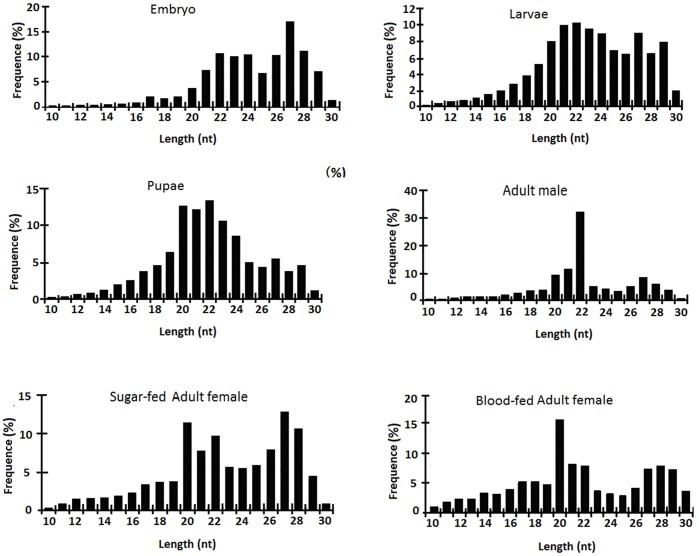
Size distribution of small RNAs derived from Illumina sequencing runs of six *Aedes albopictus* samples. Size distribution and relative frequency in each sample are shown for the small RNAs derived from embryos, larvae, pupae, adult males, sugar-fed adult females, and blood-fed adult females.

Small RNAs were sequenced from a number of developmental stages to increase the likelihood of discovering the full complement of *Ae. albopictus* miRNAs. Small RNA libraries were constructed from embryos, larvae, pupae, adult males, sugar-fed adult females and blood-fed adult females pooled at various time points after feeding. Between 10–14 million high-quality small RNA reads were obtained from each sample after Illumina sequencing and filtering out linker sequences and ambiguous reads (Figure 1). More than 50% of the reads for all six samples were ∼22 nt in length as expected for insect miRNAs. There also is an elevated population of small RNAs ∼28 nt in length in embryos, larvae and females, which may represent *piwi-interacting* RNAs (piRNAs). The comparisons of these ∼28 nt RNAs to the *Ae. albopictus* genome sequence, when available, will help the confirmation of their piRNA status and the identification of their sources. piRNAs are known to derive from and suppress repetitive sequences including transposable elements [24]. Recently piRNAs have been shown to be involved in suppression of virus infection in mosquitoes [25], [26]. Further investigation of the piRNA pathway in *Ae. albopictus* will improve our understanding of how this important vector species may defend against repetitive sequences and viruses.

**Figure 2:**
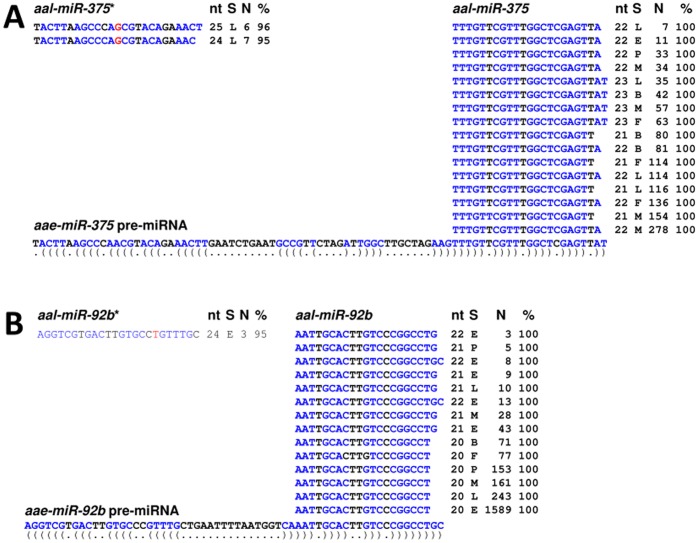
*Aedes albopictus* small RNA sequences match known *Aedes aegypti* pre-miRNAs. Two examples are shown for ***miR-375*** (**A**) and *miR-92b* (**B**). The portions of the pre-miRNA sequences (***aae-miR-375 pre-miRNA*** **and** ***aae-miR-92b pre-miRNA***) from *Ae. aegypti* with similarity to the *Ae. albopictus* reads are shown at the bottom of each image. Nucleotides in blue are those that contribute to the secondary structure of the pre-miRNA hairpin. Abbreviations: **nt**, length of small RNA read in nucleotides; **S**, sample from which the small RNAs were sequenced; **M**, adult male; **F**, adult female; **L**, larvae; **E**, embryos; **B**, blood-fed female; **N**, number of reads in each sample that showed the exact sequence; %, percentage of sequence identity between the *Ae. albopictus* small RNA read and the *Ae. aegypti* pre-miRNA. Nucleotides in red indicate sequence differences between *Ae. albopictus* and *Ae. aegypti* in the miRNA*. The 5′- to 3′-orientation of the sequences is listed from left to right in the image.

A total of 104 miRNA genes with sequence similarity to previously-described miRNAs (miRBase v17) were identified in *Ae. albopictus* (Tables S1). All but one of the 88 known *Ae. aegypti* pre-miRNA genes were recovered (Table S1; Figure 2; Li *et al*. [13]; miRBase version17). *aae-miR-1174*, the only *Ae. aegypti* miRNA that was not represented in any of the *Ae. albopictus* libraries, also is undetectable by northern blot analysis of samples from all developmental stages [27]. However, small RNAs matching aae-miR-1174* are found, and the putative *Ae. albopictus* orthologue, aal-miR-1174*, shares 21 nt identity with the mature *cqu-miR-1174* from *Cu. quinquefasciatus*. Thus, members of the miR-1174 family may have undergone an arm switch [28] with respect to its mature and star sequences in different mosquito species. All orthologues miRNAs that could be recovered according to published data were found in this study, supporting the conclusion that the approach used here is comprehensive.

### Novel miRNAs

**Figure 3:**
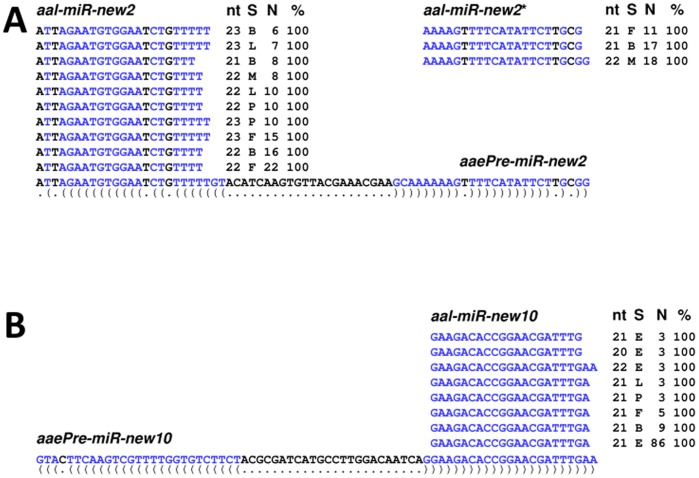
*Aedes albopictus* small RNA sequences match novel *Aedes aegypti* pre-miRNAs. Two *Ae. albopictus* miRNAs, ***aal-miR-new2*** (**A**) and ***aal-miR-new10*** (**B**), led to the discovery of novel miRNA genes in *Ae. aegypti*, designated here as ***aaePre-miR-new2*** and ***aaePre-miR-new10***, respectively. Only the most abundant reads from the *Ae. albopictus* samples are listed here. All notations are identical to Figure 2.

A total of 15 novel miRNAs were found in *Ae. albopictus* by matching small RNA reads with the *Ae. aegypti* genome sequence and performing subsequent bioinformatic confirmation (Table 1; Figure 3). The criteria set forth by Berezikov *et al*. [11] were used to ensure the authenticity of the novel miRNAs. The reliance on matching *Ae. aegypti* pre-miRNAs to confirm the authenticity of the *Ae. albopictus* miRNAs results in finding only those that are conserved between the two species. *Aedes aegypti* and *Ae. albopictus* are relatively closely-related, both belonging to the same subgenus Stegomya [29], thus it is likely that the number of *albopictus*- or *aegypti*-specific miRNAs will be low. Indeed, all of the 88 reported *Ae. aegypti* miRNA genes (miRBase v.17) were found in *Ae. albopictus*. Small RNAs that do not map to the *Ae. aegypti* genome were not investigated because genomic DNA sequencing flanking them are needed to evaluate the folding of the pre-miRNA hairpins. The presence of a pre-miRNA hairpin is a critical requirement for authentic miRNAs [11]. Such unmapped miRNAs, if present, are likely *albopictus*-specific and will be uncovered when the *Ae. albopictus* genome assembly becomes available.

**Table 1:** Novel miRNAs discovered in *Aedes albopictus*.

Name^1^	Sequence (5′-3′)	Contig number^2^	Start^3^	End^4^	Strand^5^
**Mosquito-specific**					
aal-miR-new1	AAAGCAACCGAACAATTGTCCA	25767	26526	26448	−
aal-miR-new1*	TGGCTTTTGCTTGGTAGCCTCA	25767	26526	26448	−
aal-miR-new2	ATTAGAATGTGGAATCTGTTTT	21646	21603	21512	−
aal-miR-new2*	AAAAGTTTTCATATTCTTGCG(G)^6^	21646	21603	21512	−
**Culicinae-specific**					
aal-miR-new3-1	TTCCTGACTTATACGCTTACCT	8458	2362	2439	+
aal-miR-new3-1*	TAAGTAGATAAATCAGAAAGA	8458	2362	2439	+
aal-miR-new3-2	TTCCTGACTTATACGCTTACCT	8459	21019	21096	+
aal-miR-new3-2*	TAAGTAGATAAATCAGAAAGA	8459	21019	21096	+
aal-miR-new4	TAAGCAATTTATCGTCTTGAT(G)^6^	4828	59852	59768	−
***Aedes*** **-specific**					
aal-miR-new5	AACGTGATGTATGTGAGAAGAG	3687	11751	11663	−
aal-miR-new6	TGTAGAAATGTTCGGATTTCGGCT(GAA)^6^	15327	62795	62885	+
aal-miR-new7	CAGGATTCGAAGTAGGTCAT(GCTA)^6^	21414	4023	3929	−
aal-miR-new8	GAGGACTAAGCGCATTTTTT	924	44864	44777	−
aal-miR-new9	TCCACTATTAGCCGCGAATTTGA	2411	9437	9370	−
aal-miR-new10	GAAGACACCGGAACGATTTGA	24094	20128	20040	−
aal-miR-new11	TTTGACAGTTCTGAAGATGAC	1075	3388	3473	+
aal-miR-new12	ATGGTACATTGAAGTAGGTGAG	22140	9042	8966	−
aal-miR-new13	CTTCATGATGACAACTTACACA	11123	34944	34853	−
aal-miR-new13*	TATGTTTGGTTCATACTGAT	11123	34944	34853	−
aal-miR-new14	AGGGAAGGCAGTTTGAACAGCGGG(A)^6^	25387	2495	2423	−
aal-miR-new15	AACTTTAGAAGCTTCAAGGTA	5127	29042	29129	+
aal-miR-new15*	GCCTTGACTGGTTTCCTGTT	5127	29042	29129	+

1 The nomenclature of the novel miRNAs is provisional pending further characterization. “–1”, and “–2” suffixes refer to different pre-miRNA secondary structures that produce the same mature miRNA. “Mosquito-specific” are those miRNAs found to date only in mosquitoes. “Culicinae-specific” are those miRNAs found to date only in *Aedes* and *Culex* species. “Aedes-specific” are those miRNAs found to date only in *Ae. albopictus* and *Ae. aegypti*.

2 Contig number in *Aedes aegypti* assembly version AaegL1.

3 location in contig of first nucleotide.

4 location in contig of last nucleotide.

5 5′–3′ orientation in contig (+, positive strand; –, negative strand).

6 Shown are the most abundant variants and the longest variants in parentheses.

7 “*” means “star miRNA”.

### Evolution and Expansion of Mosquito-specific miRNAs

While all mature miRNAs share sequence identity between the two *Aedes* species, there are a few cases of variation in the miRNA* sequences (Figures 2 and 3; Table S1).The *Ae. albopictus* miRNAs that matched known *Ae. aegypti* pre-miRNAs showed identical mature miRNA sequences although there are truncations at the ends as observed in previous reports [12], [13]. Three examples, miR-375*, miR92b* and miR-2946*, show one nucleotide difference between *Ae. albopictus* and *Ae. aegypti*, (Table S1, Figures 2 and 3). The majority of the miRNA genes that have homologues in non-mosquito species (Table S1) are conserved in all four mosquito species analyzed, *Ae. albopictus, Ae. aegypti*, *Cu. quinquefasciatus* and *An. gambiae.* The two exceptions are *miR-282* and *miR-927*, which are found in *Ae. aegypti* and *An. gambiae* but not in *Cu. quinquefasciatus*, indicating a loss or rapid change of these two miRNAs in the latter species (Table S3). Among the previously-known mosquito-specific miRNAs, *miR-2941* is found only in *Aedes* while *miR-1889*, *miR-2940*, and *miR-2946* are conserved in the Culicinae subfamily, which includes *Aedes* and *Culex* species (Table S1). The rest of the known mosquito miRNAs are conserved in all four mosquito species (Table S3). In contrast, only two of the 15 novel miRNAs are conserved in all four species in the three mosquito genera (Table 1, Table S1). Two additional miRNA genes are conserved in the Culicinae subfamily, and 11 are found only in *Aedes*. Furthermore, all 15 novel miRNAs appear to be unique to mosquitoes and have no sequence similarity to any known miRNAs in the miRBase (v.17) and in the sequenced genomes of *Drosophila* and other insects (data not shown). This may reflect the fact that the accumulation of entries from an ever-increasing number of organisms will exhaust the list of conserved miRNAs and newly-discovered miRNAs are likely to be lineage-specific. The current study increased the number of putative mosquito-specific miRNAs from 10 to 25 (Table S3).

Two observations support the hypothesis that the conservation and lineage-specificity of miRNAs may contain useful information to infer mosquito species phylogeny as was shown recently in other taxonomic groups [30], [31]. First, only two of the broadly-conserved miRNAs (miR-282 and miR-927) were either lost or mutated significantly in *C. quinquefasciatus*. This low rate of miRNA loss makes them good phylogenetic markers at the sub-family and genus levels. Second, the pattern of lineage-specific miRNAs is consistent with their phylogenetic relationship. For example, there are 14 *Aedes*-specific (11 novel plus 3 previously known, Table S3) and three Culicinae-specific (*miR-2941*, *miR-new3* and *miR-new4*) miRNAs. However, there are no mosquito-specific miRNAs that are shared by *Aedes* and *Anopheles* alone or *Culex* and *Anopheles* alone. Given the significant involvement of miRNAs in development, these analyses have the potential to link miRNAs to novel evolutionary and developmental features associated with a particular mosquito taxon.

### Stage-specific miRNA Expression Profiles

**Figure 4:**
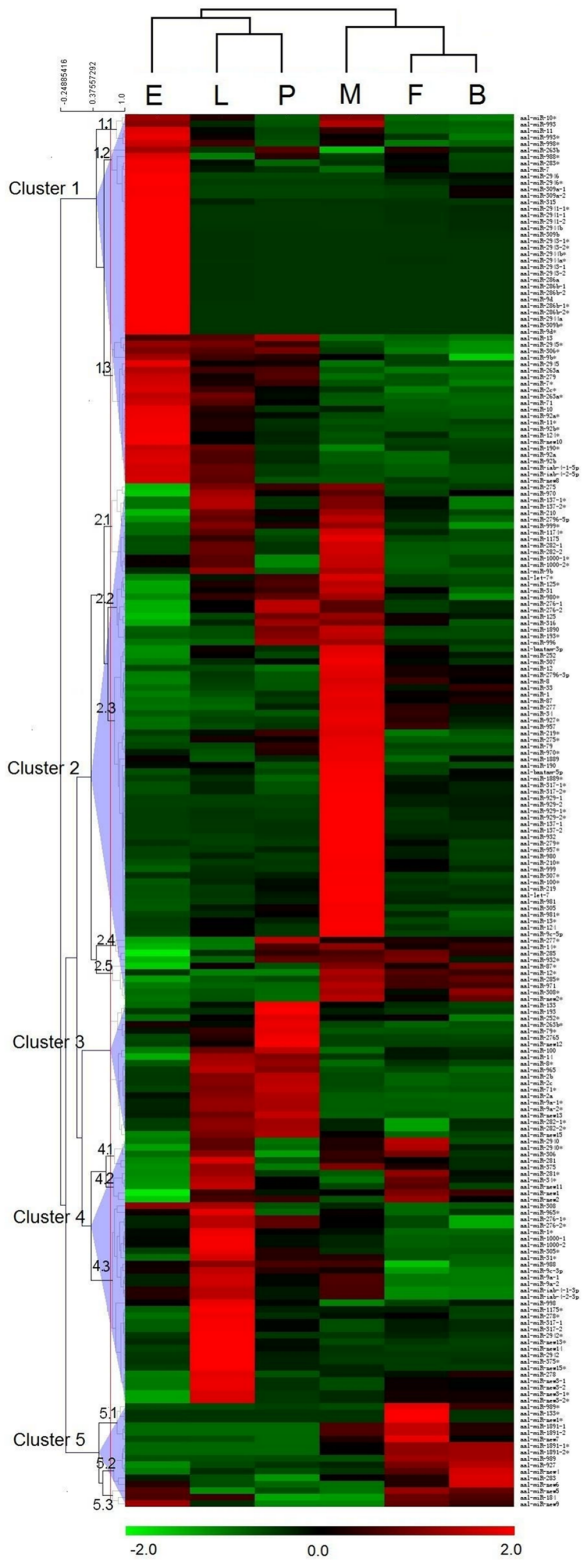
Hierarchical clustering of miRNA expression. Normalized expression profiles of 215 miRNA/miRNA*s from six different developmental stages were clustered. Stages are in columns and miRNAs in rows. Red indicates that a gene is represented highly at the stage, whereas green indicates the opposite. miRNAs with similar expression patterns cluster together. There are five Clusters (1–5) with variable numbers of sub-clusters. Abbreviations: **E**, embryos; **L**, larvae; **P**, pupae; **M**, adult males; **F**, adult females and **B**, blood-fed adult females.

The abundance of transcription from all 119 miRNA genes was evaluated for both miRNAs and miRNA*s on the basis of normalized read counts per miRNA (Table S2; Figure 4). Five clusters (1–5) and additional sub-clusters were identified. Hierarchical clustering showed that many miRNAs were embryo-specific (sub-cluster 1.2*)* including three mosquito-specific miRNAs, *miR-2941*, *miR-2943*, and *miR-2946*. The high level of miRNA accumulation in embryos is consistent with that found in *Ae. aegypti* [13], supporting the hypothesis that they may be critical for early development. miRNAs in cluster 4 are larval-specific while miRNAs in cluster 3 are either pupal-specific or expressed highly in both larval and pupal stages. A total of 71 miRNAs and miRNA*s are found predominantly in adult males (cluster 2) while 13 miRNAs and miRNA*s are abundant only in adult females (sub-cluster 5.1 and 5.2). The blood-fed female sample comprises individuals harvested at various time points after the blood meal and therefore it is likely that sensitivity in detecting dynamic changes of miRNA levels is lost. However, three small clusters of miRNAs within clusters 4 and 5 are expressed highly in sugar-fed females but at reduced levels in blood-fed females. Some miRNAs, including the novel *miRNA-new6*, showed a higher expression in blood-fed females than in sugar-fed females.

**Figure 5:**
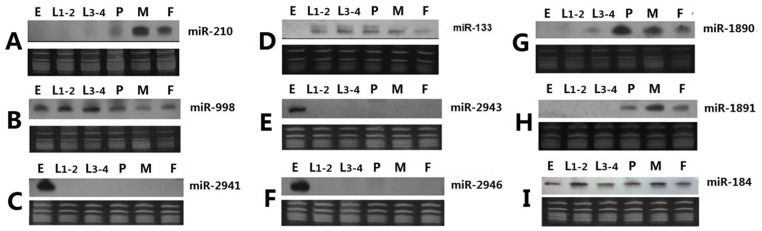
Northern blot analyses of representative miRNAs in *Aedes albopictus*. Nine representative miRNAs, ***miR-210*** (**A**), ***miR-998*** (**B**), ***miR-2941*** (**C**), ***miR-133*** (**D**), ***miR-2943*** (**E**), ***miR-2946*** (**F**), ***miR-1890*** (**G**), ***miR-1891*** (**H**) and ***miR-184*** (**I**) were subjected to northern blot analyses. The top panels are the northern results and the bottom panels are RNA gels for verification of small ribosomal and tRNA integrity and equal loading of total RNA. ssDNA size markers (19 and 23 nts, not shown) also were visualized on the RNA gel for size estimation. Fifteen micrograms of total RNA were loaded for each sample. Abbreviations: **E**, embryos; **L1–2**, mixed 1^st^ and 2^nd^ instar larvae; **L3–4**, mixed 3^rd^ and 4^th^ instar larvae; **P**, pupae; **M**, adult males and **F**, adult females.

The relative levels of six phylogenetically-conserved (*let-7*, *miR-133*, *miR-184*, *miR-210*,*miR-9a*, and *miR-998*) and six mosquito-specific (*miR-1890*, *miR-1891*, *miR-1175*, *miR-2941*, *miR-2943* and *miR- 946*) miRNAs were confirmed by northern blot analyses (Figure 5). All miRNAs tested showed accumulation in at least one of the five developmental samples. The patterns for all except *miR-9a* are consistent with the Illumina results and it is not clear at this time what caused the discrepancy with *miR-9a*. The Illumina sequencing results also are consistent with *Ae. albopictus* northern blot data reported in Zheng *et al* [27]. For example, similar to what was shown in Figure 4, the northern blot of *let-7* indicated that it accumulated mainly in developmental stages after pupation (Figure 5; Zheng *et al.* [27]). *let-7* also was shown to accumulate abundantly in the later developmental stages in *D. melanogaster, An. stephensi* and *Ae. aegypti* [12], [13], [32]. Furthermore, *let-7* plays a critical developmental regulatory role in the worm, *Caenorhabditis elegans*, by promoting the development of the 4^th^ instar larva to adult [33].

### miRNAs and Mosquito Biology

Demonstrating the functions of the 25 “mosquito-specific” miRNAs is expected to inform studies of mosquito biology and mosquito-specific adaptations. Target predictions have been used as clues to miRNA functions and such predictions for known miRNAs are available already at (www.mirbase.org). Analyses of all *Ae. albopictus* miRNAs including the 15 novel miRNAs were performed using Miranda [23] and the annotated transcripts from *Ae. aegypti* (Vectorbase). Each miRNA on average has >100 predicted targets at a stringent score of 150 (only the targets of the novel miRNAs are shown in Table S4). Gene Ontology terms associated with these targets were retrieved and enrichment analyses were performed separately for the targets of miRNAs of each of the five major expression clusters (Figure 4).The GO terms of all *Ae. aegypti* transcripts that have annotated 3′-end untranslated regions (UTR) were used as the reference and the results are shown in Figures S1 and S2. These analyses provide the basis for further functional studies to confirm the miRNA-target relationships and to investigate the functions of these miRNAs. For example, targets of miRNAs that are expressed predominantly during the embryonic stage showed significant GO term enrichment in transcription regulation, signal transduction, and cytoskeletal protein binding. The enrichment in these types of genes is consistent with expected molecular processes during embryonic development. The UTR and GO annotation is currently incomplete for the *Ae. aegypti* genome. Potential mRNA targets will not be identified unless their 3′UTRs are annotated. Nonetheless, GO terms of 6741 transcripts are available for analysis. Therefore the incompleteness of UTR and GO annotation should not significantly affect the general conclusion of the GO analysis described above, unless there is a systemic bias in the annotation of a certain class of genes.

Female mosquitoes feed on blood to acquire proteins necessary for reproduction and nutrition. Transmission of vector-borne pathogens requires blood feeding on an infected host and the blood meal also triggers a cascade of endocrinological, molecular, and physiological events that switch the adult female from host-seeking to reproduction [34], [35]. Therefore it is not unexpected to detect increases or decreases in accumulation of the majority of 119 miRNAs post blood-feeding. These miRNAs are likely important regulators of blood-meal-induced molecular changes. For example, *aae-miR-275* affects blood digestion, fluid secretion, and egg development in *Ae. Aegypti* [36]. *Aedes albopictus* has undergone a rapid expansion in its world-wide distribution and it is emerging as a vector for Dengue and Chikungunya viruses [14]. Understanding the function of a key class of gene regulators in this important mosquito vector has biological and practical significance.

## Supporting Information

Figure S1 **Color-coded gene ontology (GO) graph showing significantly-enriched GO terms describing biological processes.** Predicted targets of miRNAs within each cluster were analyzed separately. A false discovery rate (FDR) of 0.01 was used as the threshold. For each GO term, a brief description, GO number, FDR and P value were shown.
(PDF)

Figure S2 **Color-coded gene ontology (GO) graph showing significantly enriched GO terms describing molecular functions.** Predicted targets of miRNAs within each cluster (Figure 4) were analyzed separately. A false discovery rate (FDR) of 0.01 was used as the threshold. For each GO term, a brief description, GO number, FDR and P value were shown. No GO term was enriched significantly for targets of miRNAs in cluster 3 at a FDR of 0.01.
(PDF)

Table S1 Sequence, location, expression, and hairpins of miRNAs in *Aedes albopictu.*
(XLSX)

Table S2 Number of small RNA reads mapped to a miRNA/miRNA*.
(XLS)

Table S3 Distribution of mosquito-specific miRNAs as indicated by Mapmi values.
(XLSX)

Table S4 Predicted targets of the 15 novel miRNAs. Note that targets for miRNA* that showed >15 total hits in the 6 samples are also shown.
(XLS)
