# Supplementary material for: miRNA Genes of an Invasive Vector Mosquito, Aedes albopictus
Source: PLoS One. 2013 Jul 1;8(7):e67638. doi: 10.1371/journal.pone.0067638 (PMC3698096; doi:10.1371/journal.pone.0067638)
Supplement: Figure S1 — Color-coded gene ontology (GO) graph showing significantly-enriched GO terms describing biological processes. Predicted targets of miRNAs within each cluster were analyzed separately. A false discovery rate (FDR) of 0.01 was used as the threshold. For each GO term, a brief description, GO number, FDR and P value were shown. (PDF) [file pone.0067638.s001.pdf]

Figure S1. Color-coded gene ontology (GO) graph showing significantly enriched GO terms describing biological processes. Predicted targets of miRNAs within each cluster (Figure 4) were analyzed separately. A false discovery rate (FDR) of 0.01 was used as the threshold. For each GO term, a brief description, GO number, FDR and P value were shown.

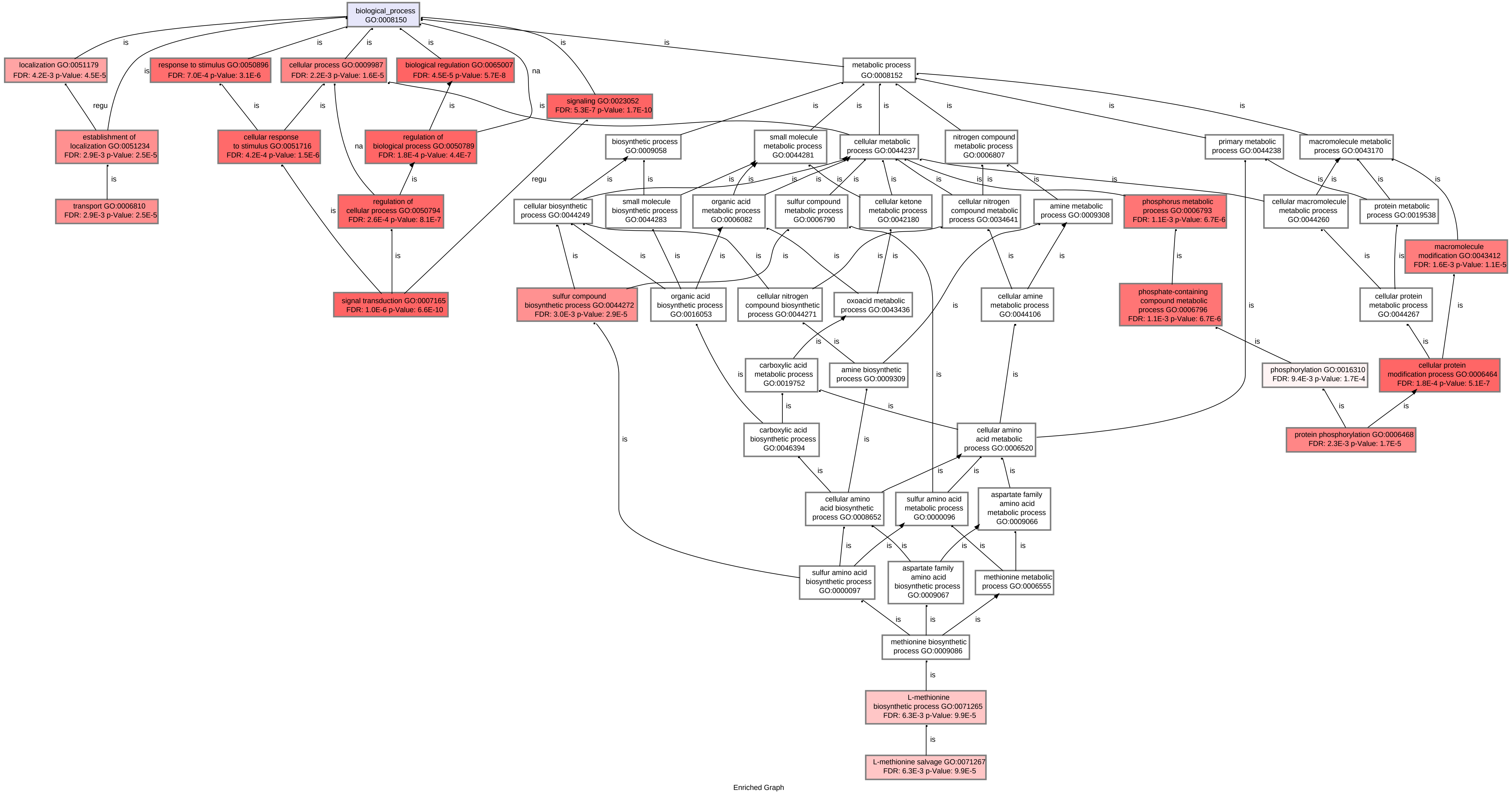
biological_process GO:0008150
localization GO:0051179 FDR: 4.2E-3 p-Value: 4.5E-5
response to stimulus GO:0050896 FDR: 7.0E-4 p-Value: 3.1E-6
cellular process GO:0009987 FDR: 2.2E-3 p-Value: 1.6E-5
biological regulation GO:0065007 FDR: 4.5E-5 p-Value: 5.7E-8
signaling GO:0023052 FDR: 5.3E-7 p-Value: 1.7E-10
metabolic process GO:0008152
establishment of localization GO:0051234 FDR: 2.9E-3 p-Value: 2.5E-5
cellular response to stimulus GO:0051716 FDR: 4.2E-4 p-Value: 1.5E-6
regulation of biological process GO:0050789 FDR: 1.8E-4 p-Value: 4.4E-7
transport GO:0006810 FDR: 2.9E-3 p-Value: 2.5E-5
regulation of cellular process GO:0050794 FDR: 2.6E-4 p-Value: 8.1E-7
signal transduction GO:0007165 FDR: 1.0E-6 p-Value: 6.6E-10
biosynthetic process GO:0009058
small molecule metabolic process GO:0044281
cellular metabolic process GO:0044237
nitrogen compound metabolic process GO:0006807
primary metabolic process GO:0044238
macromolecule metabolic process GO:0043170
cellular biosynthetic process GO:0044249
small molecule biosynthetic process GO:0044283
organic acid metabolic process GO:0006082
sulfur compound metabolic process GO:0006790
cellular ketone metabolic process GO:0042180
cellular nitrogen compound metabolic process GO:0034641
amine metabolic process GO:0009308
phosphorus metabolic process GO:0006793 FDR: 1.1E-3 p-Value: 6.7E-6
cellular macromolecule metabolic process GO:0044260
protein metabolic process GO:0019538
macromolecule modification GO:0043412 FDR: 1.6E-3 p-Value: 1.1E-5
sulfur compound biosynthetic process GO:0044272 FDR: 3.0E-3 p-Value: 2.9E-5
organic acid biosynthetic process GO:0016053
cellular nitrogen compound biosynthetic process GO:0044271
oxoacid metabolic process GO:0043436
cellular amine metabolic process GO:0044106
phosphate-containing compound metabolic process GO:0006796 FDR: 1.1E-3 p-Value: 6.7E-6
cellular protein metabolic process GO:0044267
carboxylic acid metabolic process GO:0019752
amine biosynthetic process GO:0009309
phosphorylation GO:0016310 FDR: 9.4E-3 p-Value: 1.7E-4
cellular protein modification process GO:0006464 FDR: 1.8E-4 p-Value: 5.1E-7
carboxylic acid biosynthetic process GO:0046394
cellular amino acid metabolic process GO:0006520
protein phosphorylation GO:0006468 FDR: 2.3E-3 p-Value: 1.7E-5
cellular amino acid biosynthetic process GO:0008652
sulfur amino acid metabolic process GO:0000096
aspartate family amino acid metabolic process GO:0009066
sulfur amino acid biosynthetic process GO:0000097
aspartate family amino acid biosynthetic process GO:0009067
methionine metabolic process GO:0006555
methionine biosynthetic process GO:0009086
L-methionine biosynthetic process GO:0071265 FDR: 6.3E-3 p-Value: 9.9E-5
L-methionine salvage GO:0071267 FDR: 6.3E-3 p-Value: 9.9E-5
is
regu
na
Enriched Graph

cluster 1

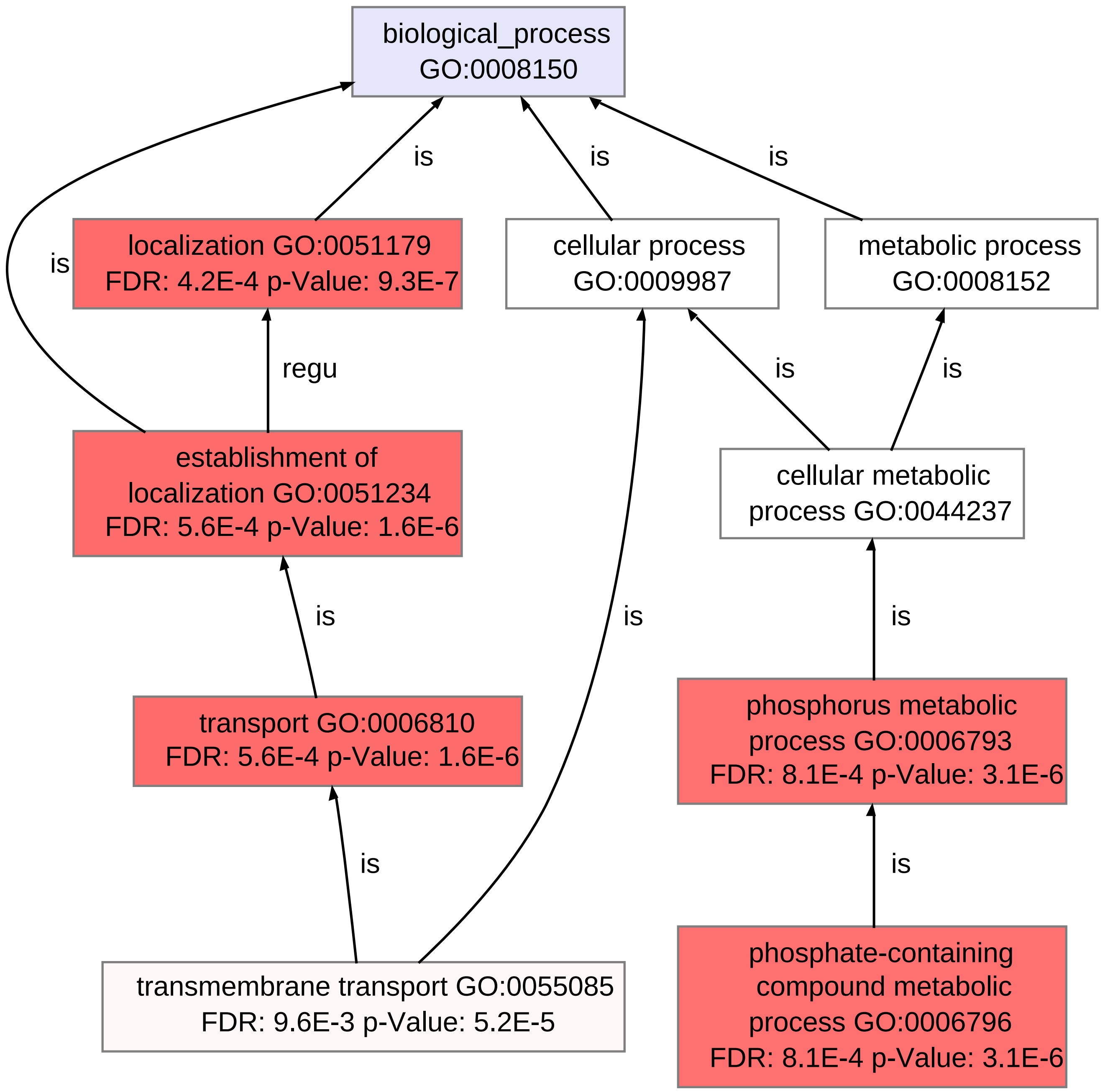

Enriched Graph

cluster 2

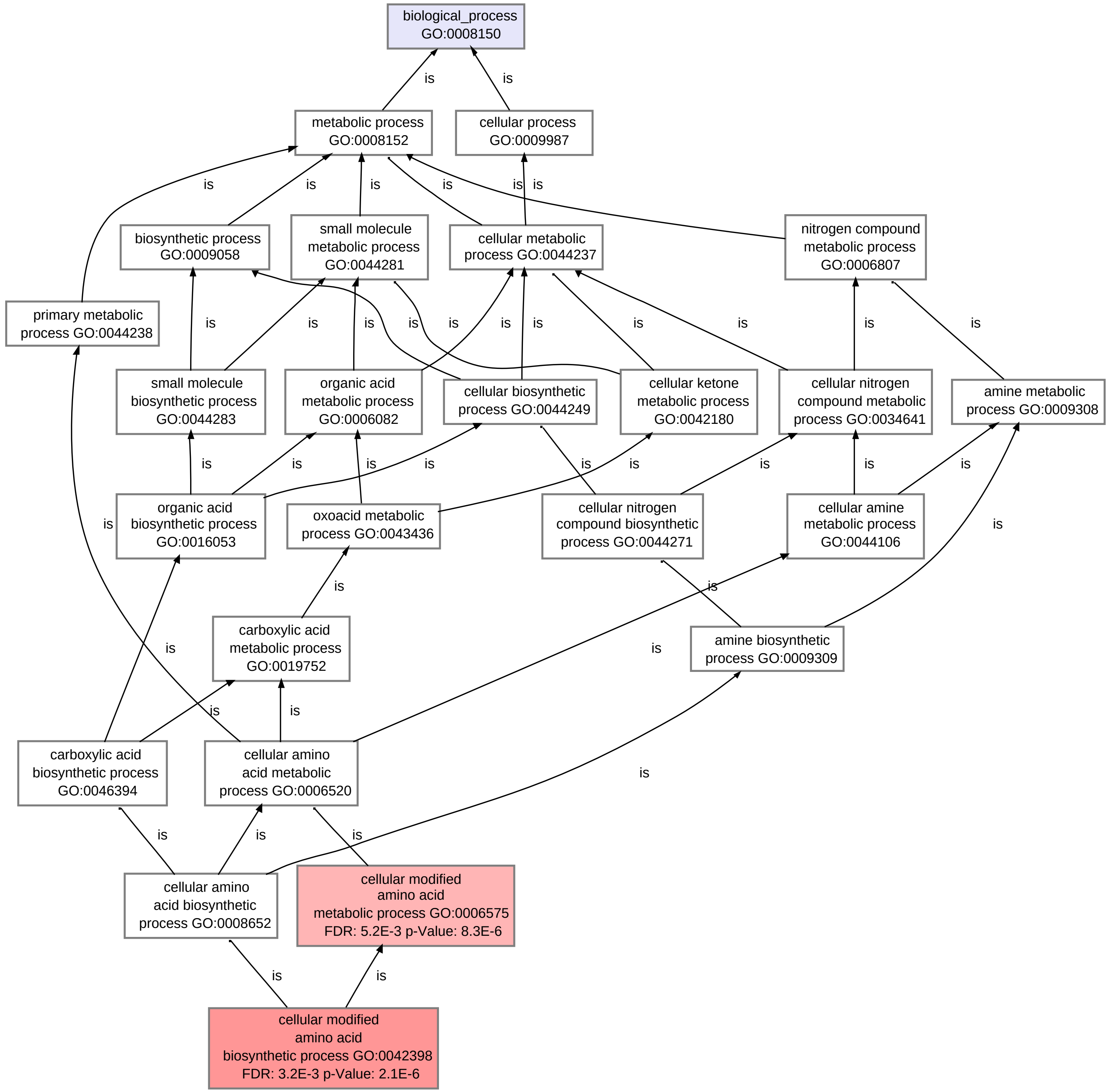

Enriched Graph

cluster 3

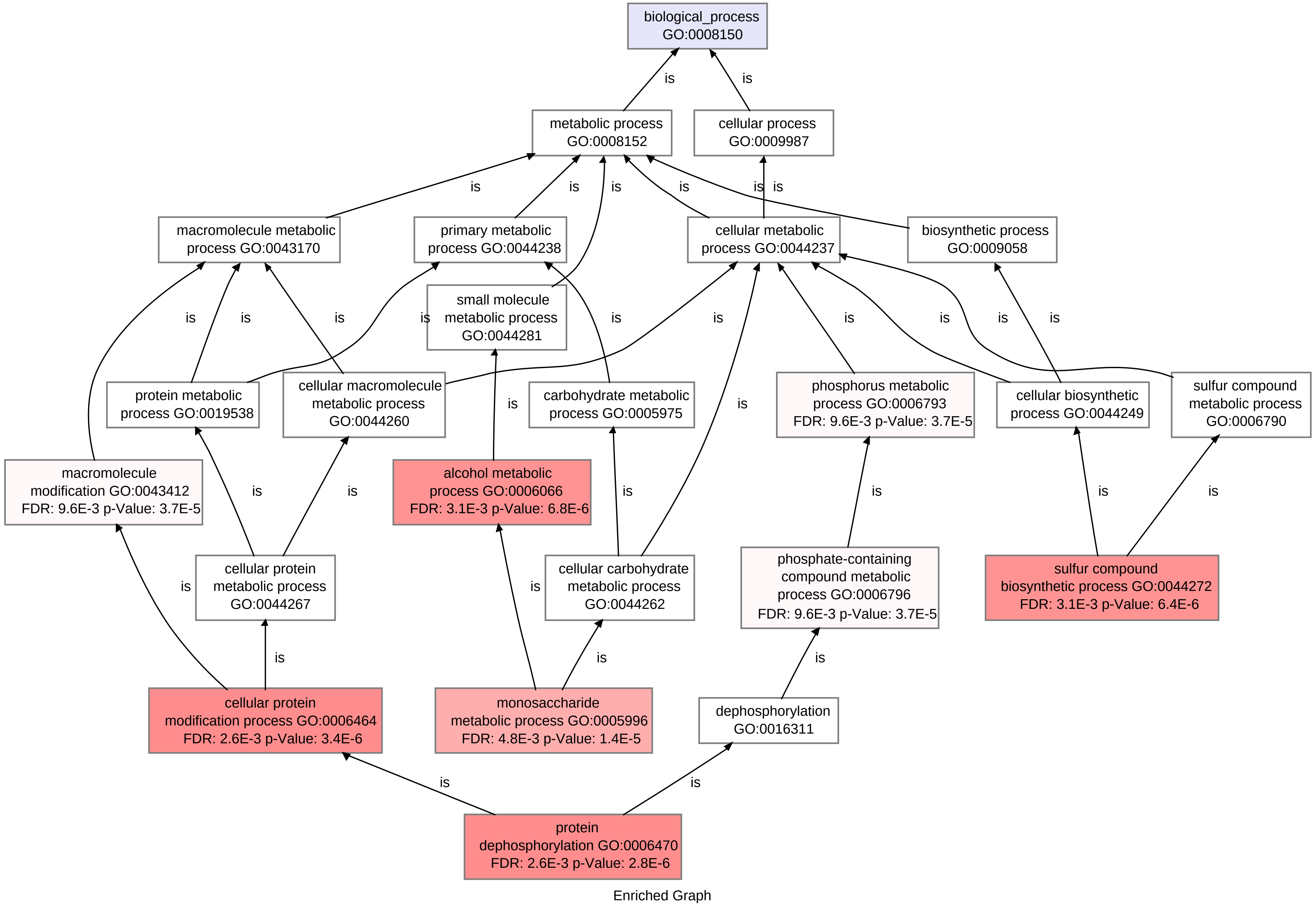
biological_process GO:0008150
metabolic process GO:0008152
cellular process GO:0009987
macromolecule metabolic process GO:0043170
primary metabolic process GO:0044238
cellular metabolic process GO:0044237
biosynthetic process GO:0009058
small molecule metabolic process GO:0044281
protein metabolic process GO:0019538
cellular macromolecule metabolic process GO:0044260
carbohydrate metabolic process GO:0005975
phosphorus metabolic process GO:0006793 FDR: 9.6E-3 p-Value: 3.7E-5
cellular biosynthetic process GO:0044249
sulfur compound metabolic process GO:0006790
macromolecule modification GO:0043412 FDR: 9.6E-3 p-Value: 3.7E-5
alcohol metabolic process GO:0006066 FDR: 3.1E-3 p-Value: 6.8E-6
cellular protein metabolic process GO:0044267
cellular carbohydrate metabolic process GO:0044262
phosphate-containing compound metabolic process GO:0006796 FDR: 9.6E-3 p-Value: 3.7E-5
sulfur compound biosynthetic process GO:0044272 FDR: 3.1E-3 p-Value: 6.4E-6
cellular protein modification process GO:0006464 FDR: 2.6E-3 p-Value: 3.4E-6
monosaccharide metabolic process GO:0005996 FDR: 4.8E-3 p-Value: 1.4E-5
dephosphorylation GO:0016311
protein dephosphorylation GO:0006470 FDR: 2.6E-3 p-Value: 2.8E-6
is
Enriched Graph

cluster 4

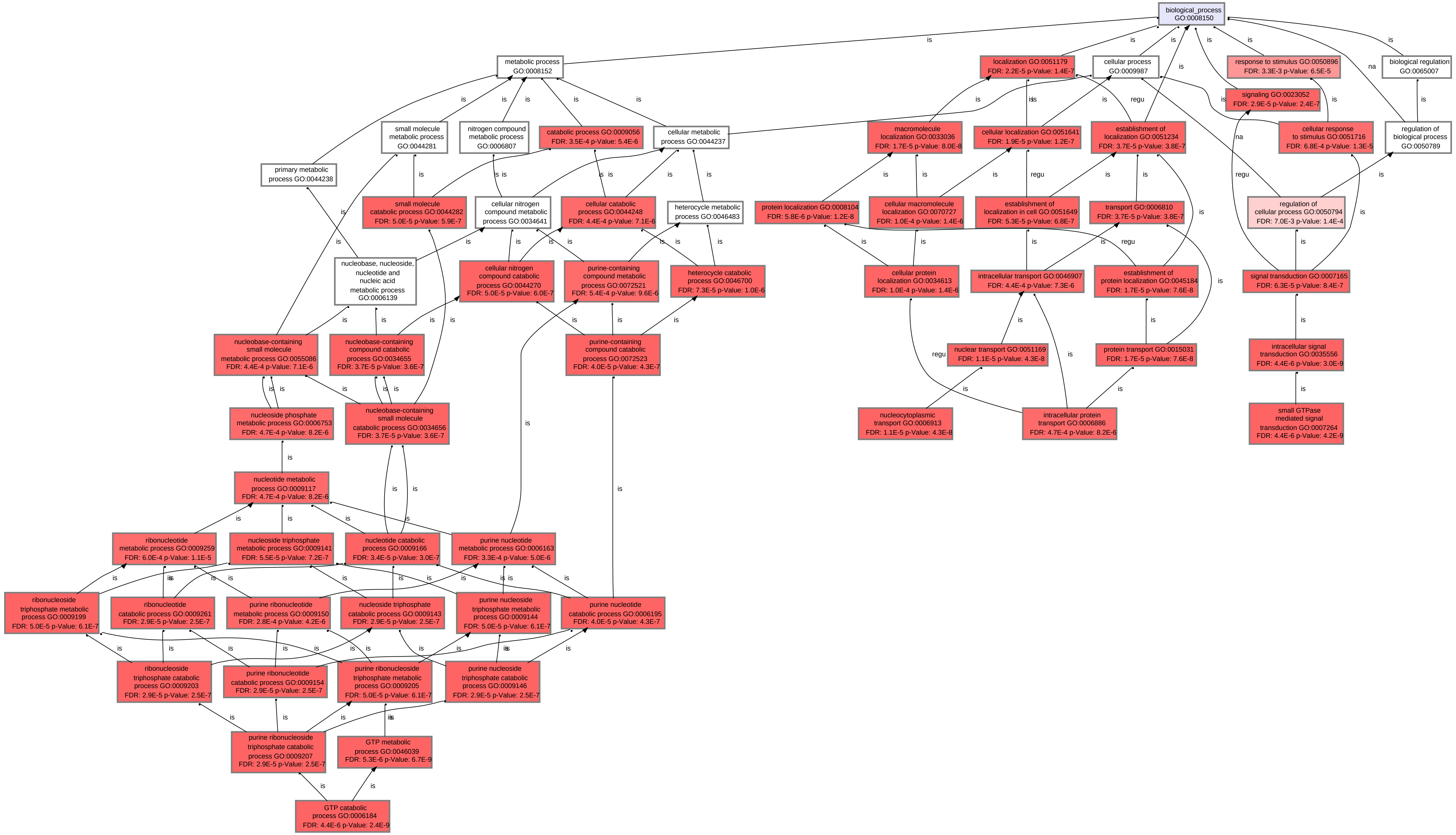

Enriched Graph

cluster 5
