# Supplementary material for: miRNA Genes of an Invasive Vector Mosquito, Aedes albopictus
Source: PLoS One. 2013 Jul 1;8(7):e67638. doi: 10.1371/journal.pone.0067638 (PMC3698096; doi:10.1371/journal.pone.0067638)
Supplement: Figure S2 — Color-coded gene ontology (GO) graph showing significantly enriched GO terms describing molecular functions. Predicted targets of miRNAs within each cluster (Figure 4) were analyzed separately. A false discovery rate (FDR) of 0.01 was used as the threshold. For each GO term, a brief description, GO number, FDR and P value were shown. No GO term was enriched significantly for targets of miRNAs in cluster 3 at a FDR of 0.01. (PDF) [file pone.0067638.s002.pdf]

Figure S2.Color-coded gene ontology (GO) graph showing significantly enriched GO terms describing molecular functions. Predicted targets of miRNAs within each cluster (Figure 4) were analyzed separately. A false discovery rate (FDR) of 0.01 was used as the threshold. For each GO term, a brief description, GO number, FDR and P value were shown. No GO term was significantly enriched for targets of miRNAs in cluster 3 at a FDR of 0.01.

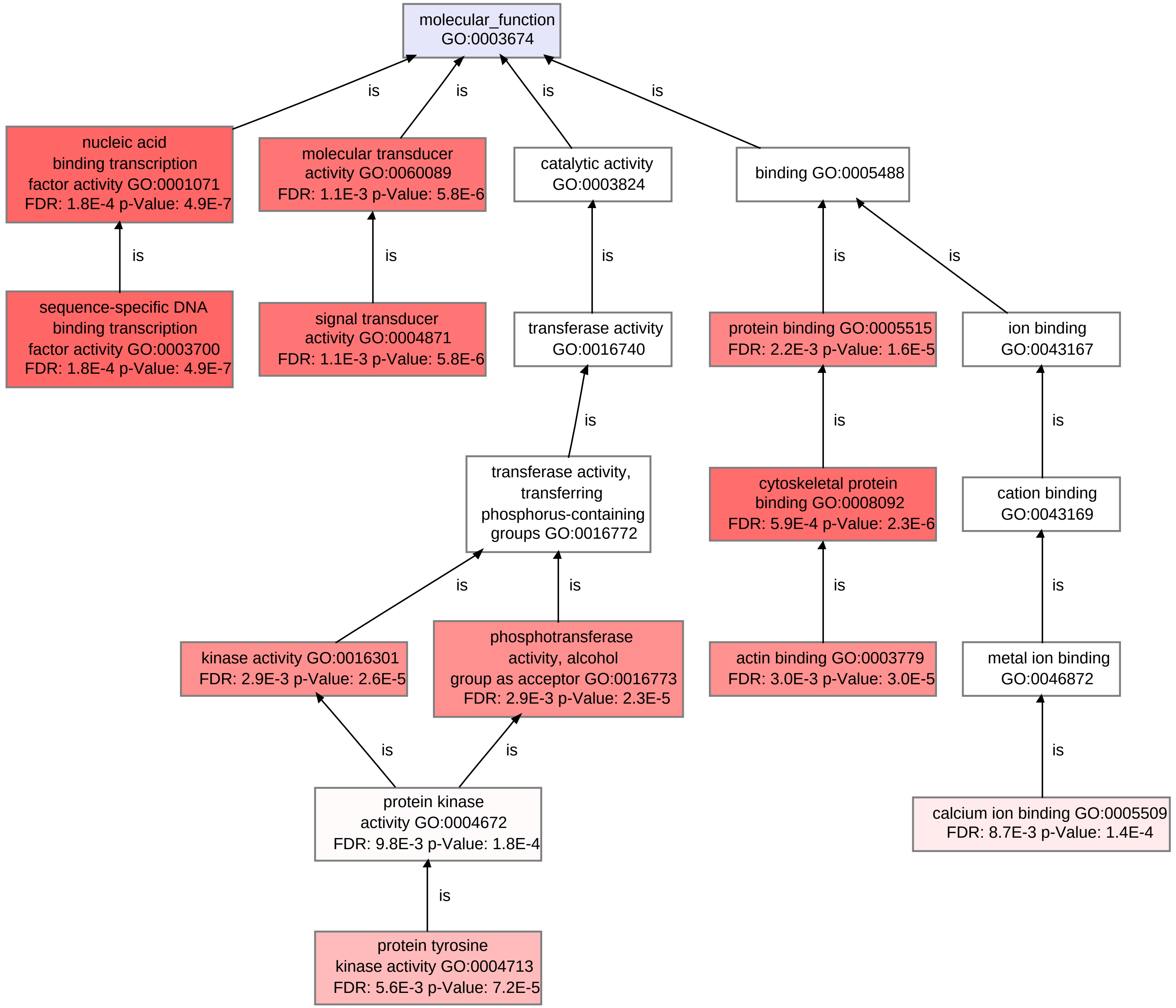

Enriched Graph

cluster 1

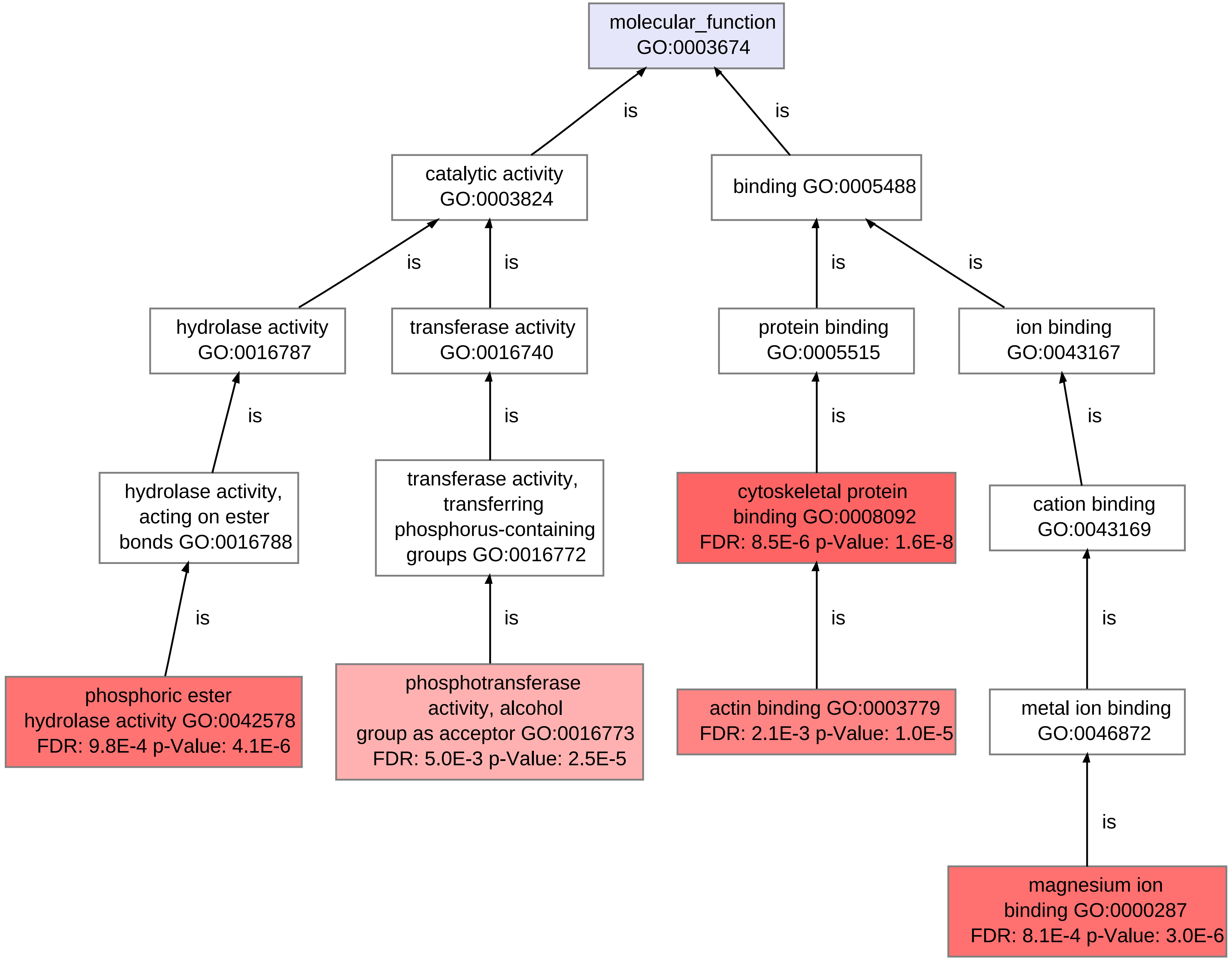

Enriched Graph

cluster 2

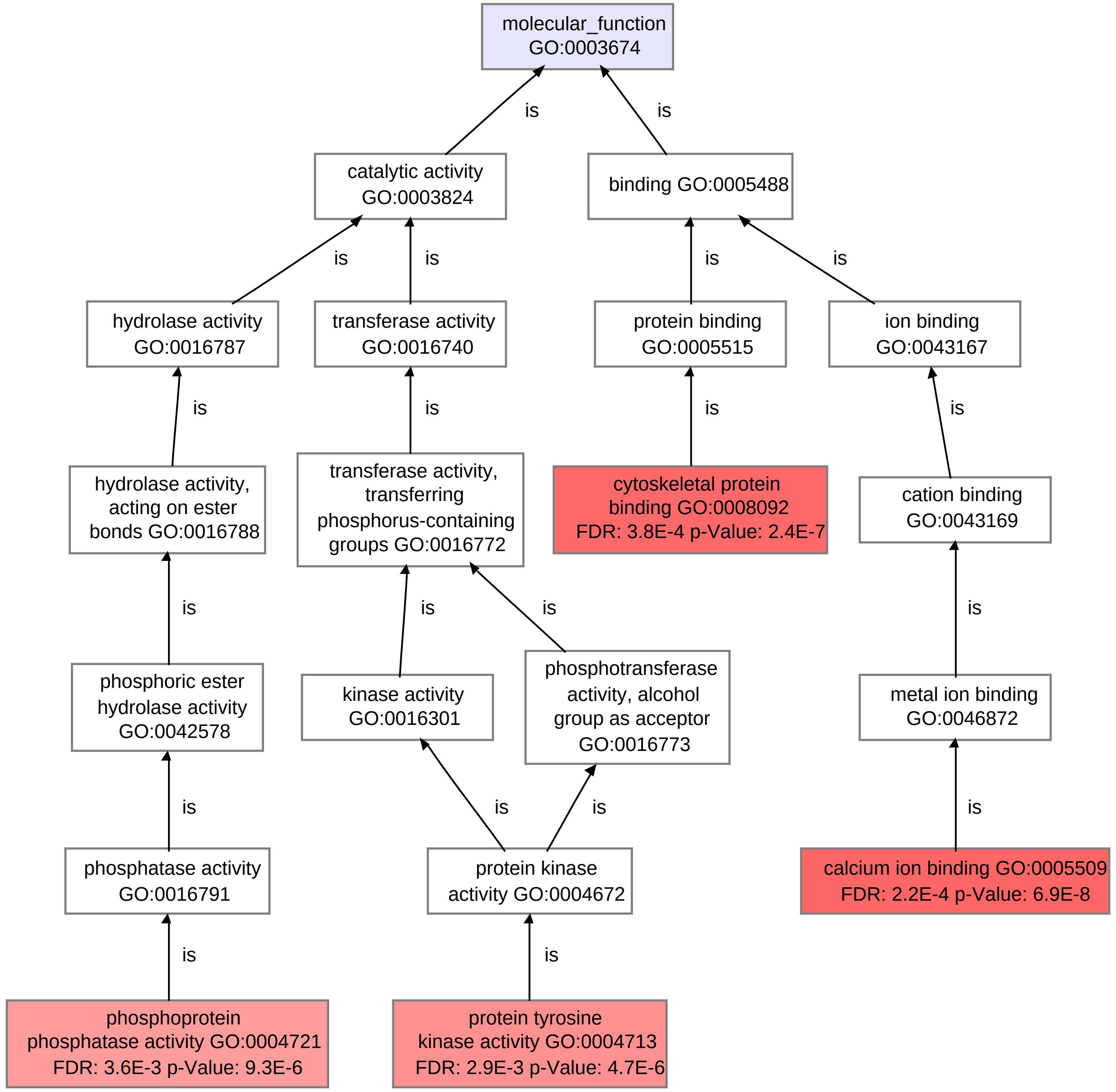

Enriched Graph

cluster 4

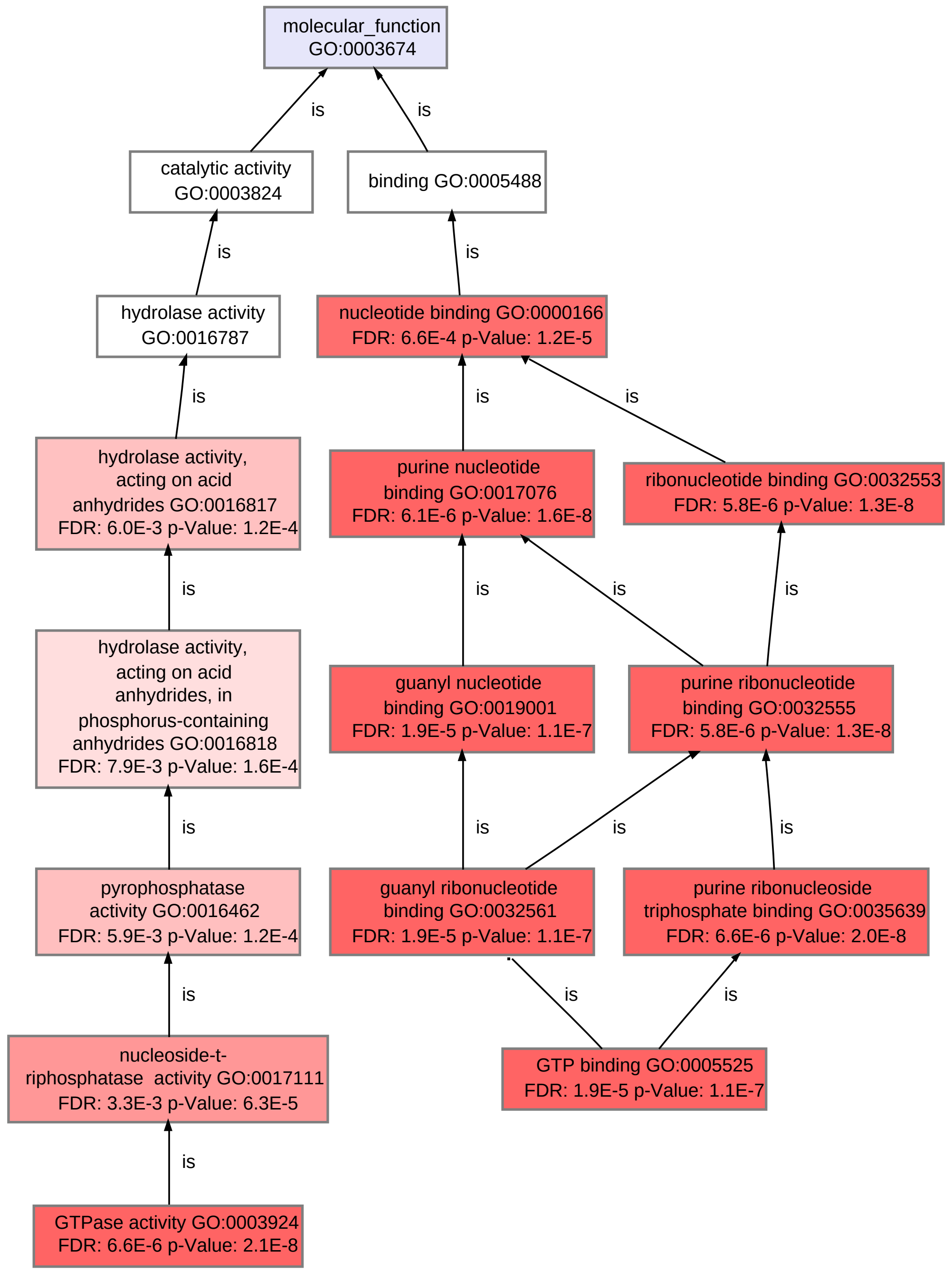

Enriched Graph

cluster 5
